# The Optimal Biological Therapy for Conversion in Patients with RAS-Wild-Type Colorectal Cancer and Unresectable Liver Metastases

**DOI:** 10.3390/cancers18213489

**Published:** 2026-10-29

**Authors:** Miljana Džunić, Ivan Petković, Ana Cvetanović, Miroslav Stojanović, Ivan Nikolić, Marina Marković, Irena Conić, Dane Krtinić

**Affiliations:** 1 Department of Oncology, Medical Faculty, University of Nis, Dr Zoran Djindjic Boulevard 81, 18108 Nis, Serbia; 2 Clinic of Oncology, University Clinical Centre of Nis, 18000 Nis, Serbia; 3 Department of Surgery, Medical Faculty, University of Nis, 18000 Nis, Serbia; 4 Clinic for Digestive Surgery, University Clinical Centre of Nis, 18000 Nis, Serbia; 5 Department of Internal Medicine, Medical Faculty, University of Novi Sad, 21000 Novi Sad, Serbia; 6 Clinic for Medical Oncology, Oncology Institute of Vojvodina, 21204 Sremska Kamenica, Serbia; 7 Department of Internal Medicine, Faculty of Medical Sciences, University of Kragujevac, 34000 Kragujevac, Serbia; 8 Centre for Medical Oncology, University Clinical Centre of Kragujevac, 34000 Kragujevac, Serbia; 9 Department of Pharmacology and Toxicology, Medical Faculty, University of Nis, 18000 Nis, Serbia

**Keywords:** colorectal cancer, metastasis, targeted treatment, clinical observation

## Abstract

**Background/Objectives:** Resection is the treatment goal in unresectable liver-limited metastatic colorectal cancer (mCRC). Anti-VEGF and anti-EGFR antibodies are effective in RAS wild-type mCRC, but the better option for conversion is unclear. We compared their efficacy and tolerability as first-line treatment. **Methods:** In this prospective, single-centre cohort study, patients with RAS-wild-type mCRC and unresectable liver metastases received cetuximab or panitumumab (anti-EGFR) or bevacizumab (anti-VEGF) with FOLFOX or FOLFIRI. We compared the overall response rate (ORR), depth of response (DoR), resection rate (RR), progression-free survival (PFS), overall survival (OS) and toxicity, using Cox regression adjusted for baseline covariates. **Results:** Of 101 patients, 51 received anti-EGFR therapy and 50 bevacizumab. Anti-EGFR therapy achieved a higher ORR (70.6% vs. 50.0%; *p* = 0.034), deeper response (47.2% vs. 33.7%; *p* = 0.004) and higher RR (66.7% vs. 46.0%; *p* = 0.036). The median PFS was longer (12.1 vs. 8.5 months; adjusted HR 0.48, 95% CI 0.29–0.78; *p* = 0.003), whereas the median OS did not differ significantly (30.4 vs. 25.5 months; *p* = 0.382). Grade 3/4 toxicity was similar (35.3% vs. 22.0%; *p* = 0.187). **Conclusions:** Anti-EGFR therapy was associated with higher response and resection rates and longer PFS and may be considered when conversion is the aim; these single-centre, non-randomised findings require confirmation in randomised studies.

## Simple Summary

For patients with colorectal cancer that has spread to the liver and cannot initially be removed by surgery, shrinking the tumour with biological drugs added to chemotherapy can make surgery possible and improve survival. In patients without mutations in the RAS genes, two types of biological drugs can be used for this purpose, anti-EGFR antibodies (cetuximab or panitumumab) or the anti-VEGF antibody bevacizumab, but it is not always clear which is more effective for this specific goal. In this single-centre cohort of 101 patients treated in Serbia, anti-EGFR therapy produced greater tumour shrinkage, allowed more patients to undergo liver surgery, and delayed disease progression for longer than bevacizumab, with a similar rate of severe side effects. These results come from a single centre and a non-randomised comparison. They suggest that anti-EGFR therapy may be considered when the goal is to convert inoperable liver metastases into operable disease, but they need to be confirmed in randomised studies.

## 1. Introduction

With more than 1.9 million new cases and over 900 000 deaths in 2022, colorectal cancer remains one of the most frequent and deadliest malignancies in the world. Metastatic colorectal cancer (mCRC), diagnosed in approximately 50% of patients, confers a dismal prognosis, with 5-year overall survival of about 14% [1].

The monoclonal antibody bevacizumab, which targets vascular endothelial growth factor (VEGF), and the antibodies directed against epidermal growth factor receptor (EGFR), cetuximab and panitumumab, have become a standard part of first-line systemic treatment, since biological therapy has been shown to improve outcomes in patients with mCRC when added to chemotherapy [2]. For patients with oligometastatic disease, prognosis can be further improved by integrating surgery of the metastatic disease into the treatment paradigm, especially when metastases are limited to the liver and/or lungs. It has been shown that patients with unresectable colorectal liver metastases (CRLMs) who undergo surgery after systemic treatment that allows resection through metastasis shrinkage have better outcomes than those treated with systemic agents alone [3]. Unlike disseminated mCRC, where the treatment goal is palliative, in unresectable liver- or lung-limited disease, the purpose of treatment is curative, aiming for conversion and resection. Therefore, the task of first-line treatment is to enable surgery through tumour shrinkage [2].

Several studies have addressed the choice of first-line biological treatment in the subpopulation with unresectable CRLM and tumours without detected mutations in the KRAS and NRAS genes (RAS wild-type), in whom either anti-EGFR or anti-VEGF treatment could be applied. The field is full of heterogeneous and contradictory results from randomised trials that included patients with different prognostic and predictive characteristics, without defined unresectability criteria, using different methodology, and without reporting long-term outcomes [4].

The aim of this study was to investigate which type of biological therapy, anti-VEGF or anti-EGFR, combined with a chemotherapy doublet, is more effective and better tolerated as first-line treatment in patients with RAS wild-type CRLM that was initially unsuitable for resection.

## 2. Materials and Methods

This prospective, single-centre cohort study enrolled patients with RAS wild-type, unresectable CRLM treated at the University Clinical Centre of Nis, Serbia, between June 2020 and January 2022.

### 2.1. Patients

Inclusion criteria were as follows: age ≥ 18 years; Eastern Cooperative Oncology Group (ECOG) performance status of 0 or 1; histologically verified adenocarcinoma of the colon or rectum with no mutations in exons 2, 3 and 4 of the KRAS and NRAS genes in the tumour specimen; multislice computed tomography (MSCT)-verified liver metastases unsuitable for initial surgical treatment. Exclusion criteria were as follows: another malignancy, except adequately treated non-melanoma skin cancer, curatively treated in situ cervical cancer or another curatively treated tumour with at least 5 years without signs of relapse; active infection; impaired renal function (creatinine clearance ≤30 mL/min); impaired hepatic function (AST and/or ALT ≥ 2.5 times the upper limit of normal [ULN] or total bilirubin ≥1.5 × ULN); and inability to understand and sign informed consent for the study.

### 2.2. Molecular Genetic Testing

The molecular genetic testing of tumour specimens was performed centrally, in the Laboratory for Medical Genetics, Medical Faculty of Nis, using the Amoy Dx KRAS/NRAS Mutation Detection Kit, which detects, viareal-time polymerase chain reaction, 19 mutations in exons 2, 3 and 4 of the KRAS gene and 13 mutations in exons 2, 3 and 4 of the NRAS gene, all relevant tomCRC.

### 2.3. Assessment of Resectability of Metastases and Surgery

The resectability of metastases was assessed by two independent, experienced hepatobiliary surgeons, initially and after every four cycles of biological therapy and chemotherapy. Liver metastases were considered unresectable if R0/R1 resection of all lesions was not possible, if the future liver remnant was less than 30% and/or if metastases were in contact with major hepatic vessels. If extrahepatic disease was present, it had to be surgically treated or resectable. The resection of liver metastases after initial systemic treatment was performed only if both surgeons gave consent for surgery. Surgery was performed 4–6 weeks after the last systemic treatment.

### 2.4. Treatment

Patients were allocated to one of the two treatment groups, bevacizumab or anti-EGFR therapy, sequentially in an alternating manner (non-randomised allocation). The anti-EGFR agent (cetuximab or panitumumab) was chosen according to its availability at the time of treatment. The chemotherapy backbone (FOLFOX4 or FOLFIRI) was chosen according to patient characteristics and physician preference, since both regimens have shown similar efficacy but different toxicity profiles in combination with biological agents [5,6,7,8]. Patients received bevacizumab 5 mg/kg, cetuximab 500 mg/m^2^ or panitumumab 6 mg/kg, combined with FOLFOX4 (oxaliplatin 85 mg/m^2^ on day 1; leucovorin 200 mg/m^2^; 5-fluorouracil 400 mg/m^2^ as a bolus and 600 mg/m^2^ as a continuous intravenous infusion on days 1 and 2) or FOLFIRI (irinotecan 180 mg/m^2^; leucovorin 200 mg/m^2^; 5-fluorouracil 400 mg/m^2^ as a bolus and 2400 mg/m^2^ as a continuous intravenous infusion). Cycles were repeated every 2 weeks. After the resection of metastases, chemotherapy without biological agents was continued for up to 6 months (12 cycles). If the metastases remained unresectable, treatment was continued until progression or unacceptable toxicity. Radiological follow-up (contrast-enhanced MSCT of the thorax, abdomen and pelvis) was performed every three months until disease progression.

### 2.5. Efficacy

The treatment response was assessed viaimaging (MSCT) and evaluated according to the Response Evaluation Criteria in Solid Tumours (RECIST), version 1.1 [9]. The overall response rate (ORR) was defined as the proportion of patients with a complete response (CR) or partial response (PR). The disease control rate (DCR) was calculated as the proportion of patients with CR, PR or stable disease (SD). The depth of response (DoR) was defined as the maximal percentage decrease in the sum of diameters of target lesions. The resection rate (RR) was defined as the percentage of patients in whom liver surgery (R0/R1) was performed. R0 and R1 resection were defined as resection with microscopically negative and positive margins, respectively. Progression-free survival (PFS) was defined as the time from the baseline radiological imaging of metastatic disease, performed the day before the start of treatment, to the date of radiological progression or death from any cause, whichever occurred first. Overall survival (OS) was defined as the time from the same baseline imaging to death from any cause.

### 2.6. Adverse Events Assessment

Treatment-related adverse events were recorded during and after every cycle of bio-chemotherapy on the basis of patient self-report and were graded according to the Common Terminology Criteria for Adverse Events (CTCAE), version 5.0.

### 2.7. Endpoints

The primary endpoint was the overall response rate (ORR); secondary endpoints were the RR, PFS, OS and treatment-related adverse events. The minimum required sample size was calculated to be 97 patients, for a statistical power of 95% and a type I error probability (α) of 0.05 (G*Power, version 3.1.9.2).

### 2.8. Statistical Analysis

Statistical analysis was performed using SPSS, version 25, and Python (statsmodels, version 0.15.0; Cox regression). Descriptive statistics were used to summarise the data. The normality of distribution was assessed using the Kolmogorov–Smirnov test. Continuous variables were compared using Student’s *t*-test (or Welch’s *t*-test when variances were unequal) or the Mann–Whitney U test, and categorical variables were compared using the chi-squared test or Fisher’s exact test when expected cell counts were below 5.

Time-to-event data were analysed using the Kaplan–Meier method, and survival was compared using the log-rank test. Patients who had not reached the endpoint at the time of analysis were censored. Median survival times are reported with 95% confidence intervals calculated using the log–log transformation. Median follow-up was estimated using the reverse Kaplan–Meier (Schemper–Smith) method, in which the event indicator is reversed so that censored patients are treated as the event of interest.

Hazard ratios (HRs) with 95% confidence intervals (CIs) for anti-EGFR therapy compared with bevacizumab (reference) were estimated using Cox proportional hazards regression with the Efron method for ties. Models were fitted without adjustment and with adjustments for age (per 10 years), sex, ECOG performance status, tumour sidedness, number and size of liver metastases, extrahepatic disease, synchronous or metachronous presentationand chemotherapy backbone (FOLFIRI vs. FOLFOX); a sensitivity model excluded the chemotherapy backbone. The proportional hazards assumption was evaluated by correlating scaled Schoenfeld residuals with time. Survival was additionally compared between patients with R0 and R1 resection, between operated patients in the two treatment groups and between patients treated with cetuximab and panitumumab (adjusted only for the chemotherapy backbone because of the limited number of events).

All tests were two-sided, and a *p*-value below 0.05 was considered statistically significant. No adjustment for multiple comparisons was made, and the subgroup analyses should be regarded as exploratory. To address the imbalance in tumour sidedness, a sensitivity analysis restricted to patients with left-sided tumours was performed with the same endpoints and models, without sidedness as a covariate. Exploratory subgroup analyses with tests for treatment-by-subgroup interactions are reported in the Supplementary Materials (Table S1, Figures S1 and S2).

### 2.9. Use of Artificial Intelligence

During the preparation of this work, the authors used Claude (Anthropic, San Francisco, CA, USA; model Claude Sonnet 5.5, accessed in October 2026) to improve the language and grammar of the text, to format the tables, figures and reference list according to the journal style, to draft and revise parts of the Discussion, to write analysis code (Python, statsmodels) for the survival and subgroup analyses and to produce the figures and the graphical abstract. The study data, the choice of statistical methods, the interpretation of the results and the conclusions are the authors’ own. All AI-generated text, code output and references were checked, verified and edited by the authors, who take full responsibility for the content of the publication. No AI tool was used to generate or alter the study data.

## 3. Results

Of the 107 patients initially enrolled, six received fewer than four treatment cycles and were excluded from the analysis. The cohort therefore included 101 patients: 51 were treated with anti-EGFR antibodies (29 with cetuximab and 22 with panitumumab; one patient who received both agents sequentially is counted in the cetuximab group here, but in the panitumumab group in Section 3.7) and 50 with bevacizumab.

### 3.1. Characteristics of Treatment Groups

The treatment groups were well balanced with respect to sex (*p* = 0.94), age (*p* = 0.97), ECOG performance status (*p* = 0.06), comorbidities (*p* = 0.77), number of liver metastases (*p* = 0.36), sum of diameters of target lesions (*p* = 0.13), size of metastases (*p* = 0.49), synchronous versus metachronous metastases (*p* = 0.85), involvement of other organs (*p* = 0.56) and number of preoperative treatment cycles received (*p* = 0.10). Significant differences were observed in the tumour sidedness (left- versus right-sided; *p* = 0.002) and in the chemotherapy backbone (*p* < 0.001) (Table 1).

### 3.2. Treatment Response and Liver Resection

The anti-EGFR group achieved a significantly higher objective response rate (ORR: 70.6% vs. 50.0%; *p* = 0.034) and a greater depth of response (DoR: 47.20 ± 22.79% vs. 33.67 ± 25.27%; *p* = 0.004), whereas the disease control rate (DCR) did not differ significantly (80.4% vs. 66.0%; *p* = 0.102). The resection rate was also significantly higher in the anti-EGFR group (66.7% vs. 46.0%; *p* = 0.036) (Table 2).

### 3.3. Resection Quality and Survival in Operated Patients

Among the 57 patients who underwent liver resection (34 in the anti-EGFR and 23 in the bevacizumab group), R0 resection was achieved in 45 (78.9%) and R1 resection in 12 (21.1%). The R1 rate did not differ between the anti-EGFR and bevacizumab groups (7/34 [20.6%] vs. 5/23 [21.7%]; *p* = 1.00) (Table 2). Median PFS did not differ significantly between patients with R0 and R1 resection (12.0 vs. 18.1 months; HR 0.66, 95% CI 0.34–1.31; *p* = 0.236), nor did median OS (37.1 vs. 38.5 months; HR 0.91, 95% CI 0.43–1.94; *p* = 0.806).

Among patients who underwent liver resection, anti-EGFR treatment showed a trend towards longer PFS compared with bevacizumab (median 14.4 vs. 11.0 months; HR 0.59, 95% CI 0.33–1.04; *p* = 0.065), whereas OS was similar (median 37.1 vs. 39.7 months; HR 1.06, 95% CI 0.56–2.03; *p* = 0.850). After resection, progression or recurrence was recorded in 27 of 34 anti-EGFR patients (79.4%) and in all 23 bevacizumab patients (*p* = 0.034); the site of first progression is shown in Supplementary Table S2.

### 3.4. Survival Analysis in the Anti-EGFR and Bevacizumab Groups

**Figure 1:**
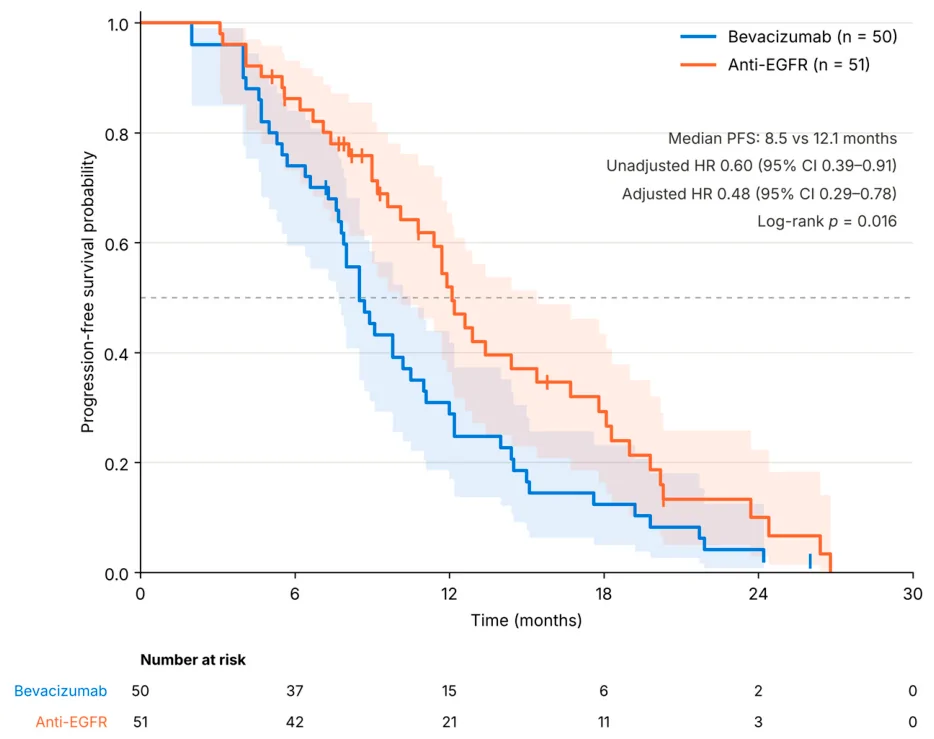
Kaplan–Meier curves of progression-free survival in patients with RAS-wild-type colorectal cancer and unresectable liver metastases treated with anti-EGFR agents versus bevacizumab. Shaded areas represent 95% confidence intervals; vertical ticks indicate censored patients. Numbers at risk are shown below the plot. Median PFS was 12.1 months (95% CI 10.1–15.4) with anti-EGFR agents versus 8.5 months (95% CI 7.7–10.5) with bevacizumab (log-rank *p* = 0.016). The adjusted hazard ratio is reported in Table 3.

At the time of analysis, progression or death had occurred in 89 of 101 patients (41 of 51 in the anti-EGFR group and 48 of 50 in the bevacizumab group). Median PFS was significantly longer in the anti-EGFR group than in the bevacizumab group (12.1 months, 95% CI 10.1–15.4, vs. 8.5 months, 95% CI 7.7–10.5; log-rank *p* = 0.016) (Figure 1). The estimated 12-month PFS rates were 51.9% and 28.8%, respectively. At 24 months, 10.0% of patients in the anti-EGFR group and 4.1% in the bevacizumab group remained free of progression.

**Table 3:** Cox proportional hazards models for progression-free and overall survival: anti-EGFR therapy versus bevacizumab (reference).

*p*	HR (95% CI)	Model	Endpoint
0.017	0.60 (0.39–0.91)	Unadjusted	PFS (89 events)
0.003	0.48 (0.29–0.78)	Adjusted for all covariates *	
0.006	0.53 (0.34–0.84)	Adjusted, excluding chemotherapy backbone	
0.380	0.81 (0.50–1.30)	Unadjusted	OS (70 events)
0.141	0.64 (0.36–1.16)	Adjusted for all covariates *	
0.206	0.71 (0.42–1.21)	Adjusted, excluding chemotherapy backbone	

HR, hazard ratio; CI, confidence interval; EGFR, epidermal growth factor receptor; PFS, progression-free survival; OS, overall survival. HR below 1 favours anti-EGFR therapy. * Adjusted for age, sex, ECOG performance status, tumour sidedness, number and size of liver metastases, extrahepatic disease, synchronous/metachronous presentation and chemotherapy backbone (FOLFIRI vs. FOLFOX).

**Figure 2:**
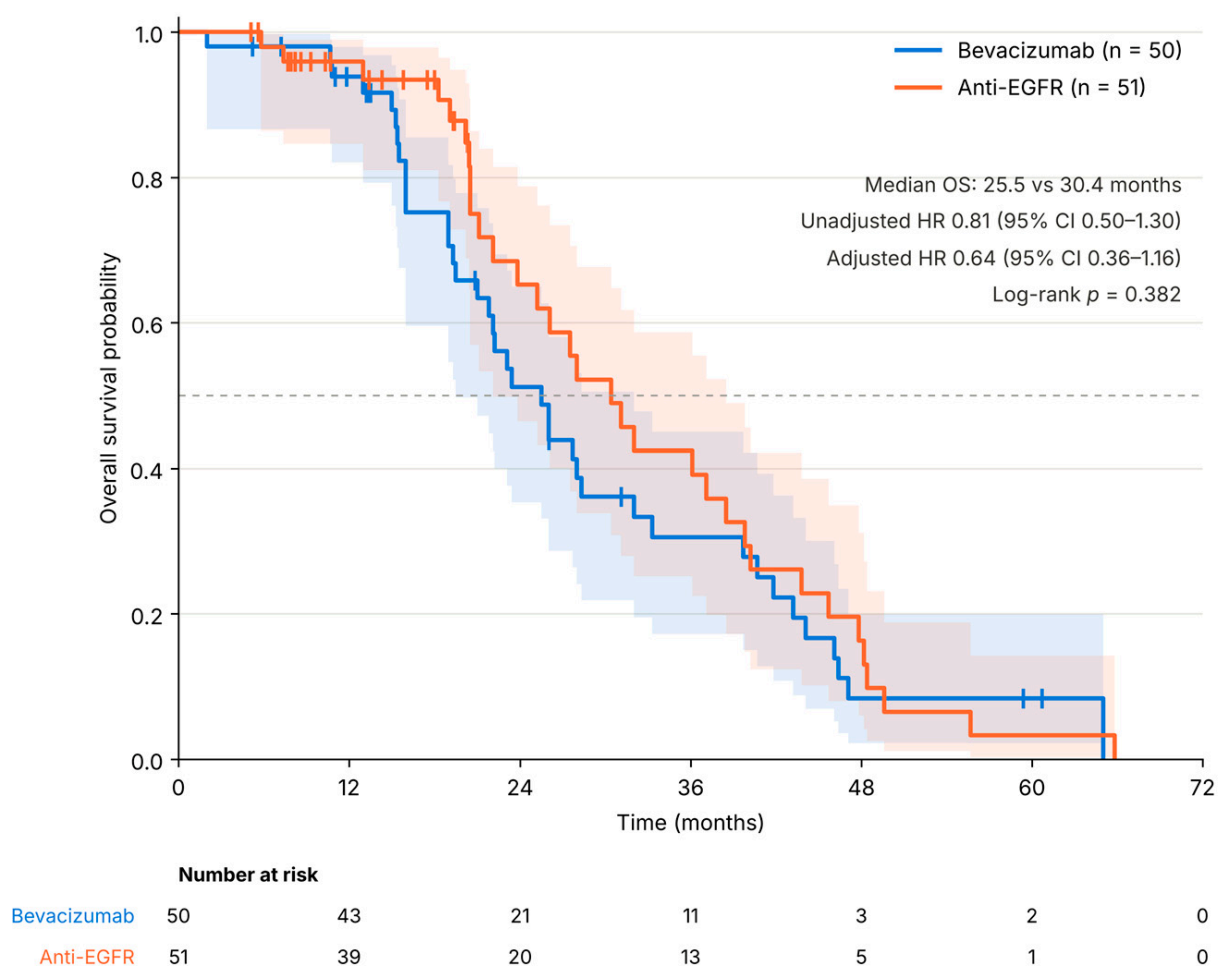
Kaplan–Meier curves of overall survival in patients with RAS-wild-type colorectal cancer and unresectable liver metastases treated with anti-EGFR agents versus bevacizumab. Shaded areas represent 95% confidence intervals; vertical ticks indicate censored patients. Numbers at risk are shown below the plot. Median OS was 30.4 months (95% CI 22.1–38.5) with anti-EGFR agents versus 25.5 months (95% CI 19.5–32.0) with bevacizumab (log-rank *p* = 0.382). The adjusted hazard ratio is reported in Table 3.

Death had occurred in 70 of 101 patients (32 in the anti-EGFR group and 38 in the bevacizumab group). Median OS was 30.4 months (95% CI 22.1–38.5) in the anti-EGFR group and 25.5 months (95% CI 19.5–32.0) in the bevacizumab group; the difference was not statistically significant (log-rank *p* = 0.382) (Figure 2). Estimated OS rates at 12, 24 and 36 months were 95.9%, 65.2% and 42.4% in the anti-EGFR group and 93.8%, 51.2% and 30.6% in the bevacizumab group. For the entire cohort, the median follow-up, estimated using the reverse Kaplan–Meier method, was 59.4 months.

### 3.5. Cox Proportional Hazards Models

Model description. Hazard ratios (HRs) for anti-EGFR therapy compared with bevacizumab (reference) were estimated using Cox proportional hazards regression, first without adjustment and then with an adjustment for nine baseline covariates: age (per 10 years), sex, ECOG performance status (1 vs. 0), tumour sidedness (right vs. left), number of liver metastases (≥5 vs. 1–4), size of metastases (>5 vs. ≤5 cm), extrahepatic disease, synchronous or metachronous presentation and chemotherapy backbone (FOLFIRI vs. FOLFOX). The PFS and OS models were based on 89 and 70 events, respectively. Because only two patients in the bevacizumab group received FOLFIRI, a sensitivity model without the chemotherapy backbone was also fitted.

Progression-free survival. Anti-EGFR therapy was associated with a lower risk of progression or death than bevacizumab in the unadjusted model (HR 0.60, 95% CI 0.39–0.91; *p* = 0.017). The association persisted after full adjustment (HR 0.48, 95% CI 0.29–0.78; *p* = 0.003) and in the model without the chemotherapy backbone (HR 0.53, 95% CI 0.34–0.84; *p* = 0.006) (Table 3).

**Figure 3:**
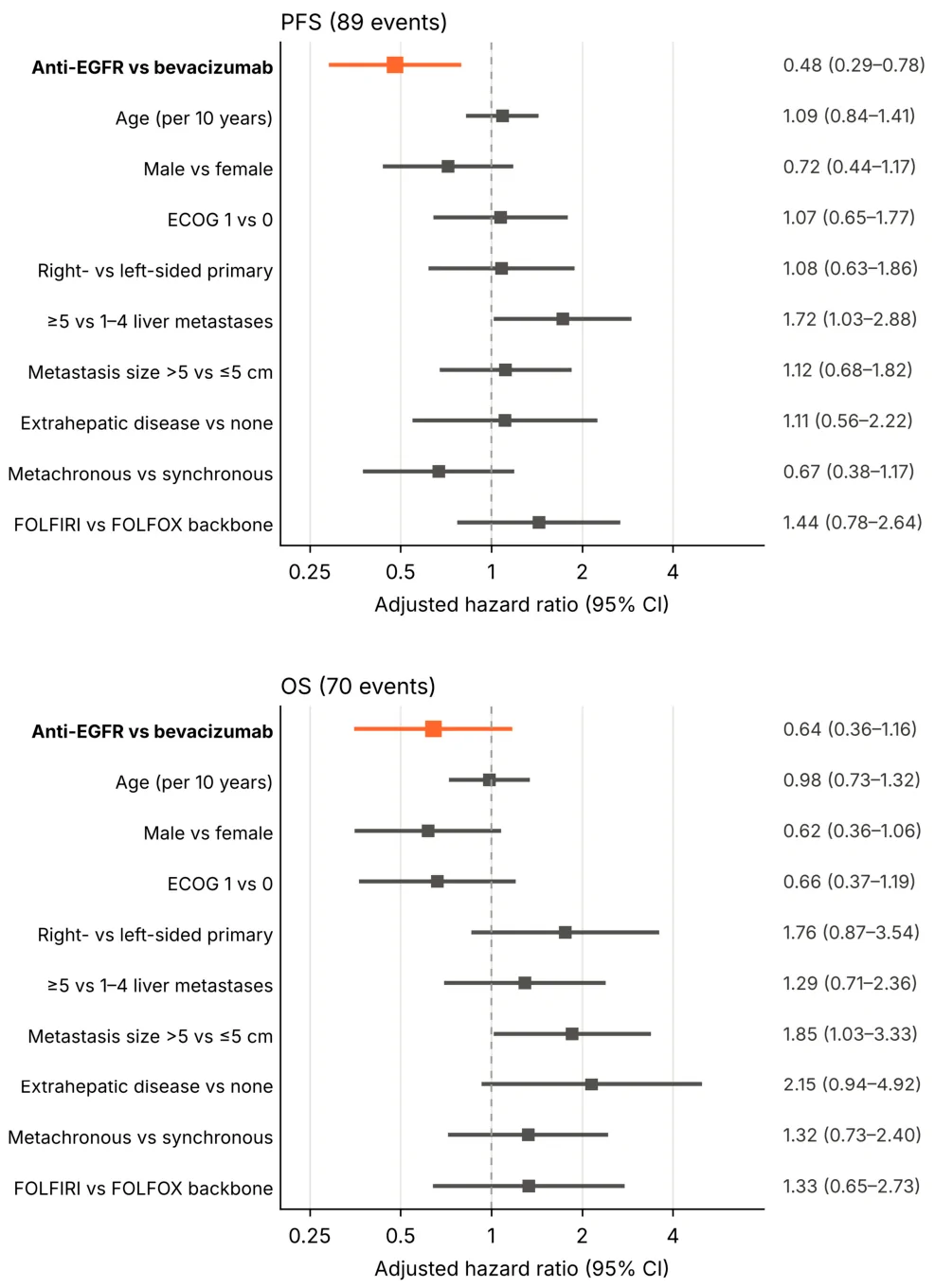
Forest plot of the multivariable Cox proportional hazards analysis of progression-free survival and overall survival. Adjusted hazard ratios (HRs) with 95% confidence intervals for anti-EGFR therapy versus bevacizumab and for baseline covariates. The model was adjusted for age, sex, ECOG performance status, tumour sidedness, number and size of liver metastases, extrahepatic disease, timing of metastases (synchronous vs. metachronous) and chemotherapy backbone. Squares indicate point estimates and horizontal lines 95% confidence intervals. The vertical dashed line represents HR = 1; HR < 1 favours anti-EGFR therapy. Upper panel, PFS (89 events; adjusted HR for anti-EGFR therapy 0.48, 95% CI 0.29–0.78); lower panel, OS (70 events; adjusted HR 0.64, 95% CI 0.36–1.16).

Overall survival. The unadjusted HR for death was 0.81 (95% CI 0.50–1.30; *p* = 0.380). The fully adjusted HR was 0.64 (95% CI 0.36–1.16; *p* = 0.141), and the HR without the chemotherapy backbone was 0.71 (95% CI 0.42–1.21; *p* = 0.206), indicating a trend in favour of anti-EGFR therapy that did not reach statistical significance (Table 3, Figure 3).

Model assumptions. An assessment of the proportional hazards assumption using scaled Schoenfeld residuals suggested that the effect of anti-EGFR therapy on PFS was not entirely constant over time (*p* = 0.026), whereas no such deviation was found for OS (*p* = 0.111). Given the limited number of events relative to the number of covariates, particularly for OS, the adjusted estimates should be interpreted with caution.

### 3.6. Prognostic Factors in the Multivariable Cox Analysis

The multivariable Cox models are shown in Figure 3. For PFS, anti-EGFR therapy remained independently associated with a lower risk of progression or death after an adjustment for all covariates (HR 0.48, 95% CI 0.29–0.78). Among the other covariates, only the presence of five or more liver metastases was significantly associated with worse PFS (HR 1.72, 95% CI 1.03–2.88). Age, sex, ECOG performance status, tumour sidedness, metastasis size, extrahepatic disease, timing of metastases and chemotherapy backbone were not significantly associated with PFS, although FOLFIRI showed a numerically higher hazard than FOLFOX (HR 1.44, 95% CI 0.78–2.64).

For OS, anti-EGFR therapy showed a favourable trend that did not reach statistical significance (adjusted HR 0.64, 95% CI 0.36–1.16). Metastases larger than 5 cm were independently associated with shorter OS (HR 1.85, 95% CI 1.03–3.33). Extrahepatic disease (HR 2.15, 95% CI 0.94–4.92) and right-sided primary tumours (HR 1.76, 95% CI 0.87–3.54) also showed higher hazards, but the confidence intervals included 1. Male sex was associated with a lower hazard of death that was borderline (HR 0.62, 95% CI 0.36–1.06). The wide confidence intervals for several covariates reflect the limited number of events (89 for PFS and 70 for OS).

Sensitivity analysis in left-sided tumours. Left-sided tumours were present in 78 patients (46 in the anti-EGFR and 32 in the bevacizumab group). In this subgroup, the ORR (69.6% vs. 53.1%; *p* = 0.159), DCR (80.4% vs. 68.8%; *p* = 0.288) and resection rate (67.4% vs. 53.1%; *p* = 0.241) were numerically higher with anti-EGFR therapy, and the depth of response was greater (47.4 ± 23.4% vs. 32.5 ± 19.6%; *p* = 0.011). Median PFS was 12.2 vs. 9.1 months (log-rank *p* = 0.044; unadjusted HR 0.61, 95% CI 0.38–0.99; adjusted HR 0.53, 95% CI 0.30–0.94; *p* = 0.031), and median OS was 32.0 vs. 26.0 months (log-rank *p* = 0.566; unadjusted HR 0.85, 95% CI 0.50–1.45; adjusted HR 0.57, 95% CI 0.29–1.13; *p* = 0.107). The results were therefore consistent with the main analysis (Supplementary Table S3).

### 3.7. Comparison of Cetuximab and Panitumumab Within the Anti-EGFR Group

Of the 51 patients treated with anti-EGFR antibodies, 28 received cetuximab and 23 panitumumab (one patient who received cetuximab followed by panitumumab owing to an allergic reaction was included in the panitumumab group) (Table 4). The two groups were similar in age (58.8 ± 12.6 vs. 62.9 ± 7.7 years; *p* = 0.156), ECOG performance status, comorbidity, sidedness, number and size of liver metastases and the presence of other organ involvement (all *p* > 0.10). Men were more frequent in the panitumumab group (82.6% vs. 53.6%; *p* = 0.039), and metachronous metastases were numerically more frequent in the panitumumab group (34.8% vs. 14.3%; *p* = 0.107). All patients treated with panitumumab received FOLFOX, whereas 71.4% of patients treated with cetuximab received FOLFIRI (*p* < 0.001). The number of therapy cycles (6.1 ± 2.5 vs. 6.9 ± 2.8; *p* = 0.292) did not differ between the groups.

The overall response rate (71.4% vs. 69.6%; *p* = 1.000), disease control rate (78.6% vs. 82.6%; *p* = 1.000) and depth of response (44.8 ± 21.8% vs. 50.0 ± 24.1%; *p* = 0.475) were comparable. Resection was performed in 17 patients (60.7%) treated with cetuximab and 17 patients (73.9%) treated with panitumumab (*p* = 0.381).

Median PFS was 12.1 months (95% CI 8.1–14.4) with cetuximab and 12.9 months (95% CI 9.6–20.3) with panitumumab (log-rank *p* = 0.037; unadjusted HR for panitumumab vs. cetuximab 0.48, 95% CI 0.24–0.97). After an adjustment for chemotherapy backbone, the difference was no longer significant (HR 0.56, 95% CI 0.21–1.53). Median OS was 27.5 months (95% CI 20.5–37.1) with cetuximab and 32.0 months (95% CI 20.4–48.2) with panitumumab (log-rank *p* = 0.141; unadjusted HR 0.57, 95% CI 0.27–1.21; adjusted HR 0.57, 95% CI 0.19–1.66). Because the chemotherapy backbone differed completely between the agents, these comparisons should be interpreted with caution. Within the anti-EGFR group, the ORR (67.7% vs. 75.0%; *p* = 0.755) and resection rate (71.0% vs. 60.0%; *p* = 0.545) did not differ significantly between FOLFOX and FOLFIRI (Supplementary Table S4).

### 3.8. Adverse Events

Any adverse event occurred in 47 of 51 patients (92.2%) treated with anti-EGFR therapy and in 35 of 50 patients (70.0%) treated with bevacizumab (*p* = 0.005) (Table 5 and Table 6). Grade 3/4 adverse events were recorded in 18 patients (35.3%) in the anti-EGFR group and 11 patients (22.0%) in the bevacizumab group, and this difference was not statistically significant (*p* = 0.187).

The toxicity profiles of the two treatments differed in a manner consistent with their mechanisms of action. A skin rash was the most frequent adverse event in the anti-EGFR group (86.3% vs. 2.0%; *p* < 0.001), and a grade 3/4 rash occurred in 10 patients (19.6%) treated with anti-EGFR therapy and in none treated with bevacizumab (*p* = 0.001). Diarrhoea was also more frequent with anti-EGFR therapy (35.3% vs. 10.0%; *p* = 0.004), although grade 3/4 diarrhoea was uncommon (3.9% vs. 0%; *p* = 0.495). Stomatitis (9.8% vs. 0%; *p* = 0.056), hand–foot syndrome (5.9% vs. 0%; *p* = 0.243) and paronychia (3.9% vs. 0%; *p* = 0.495) occurred only in anti-EGFR-treated patients, but the differences were not statistically significant.

In contrast, hypertension was recorded only in patients treated with bevacizumab (38.0% vs. 0%; *p* < 0.001), and grade 3/4 hypertension occurred in four patients (8.0%; *p* = 0.056). Polyneuropathy (20.0% vs. 2.0%; *p* = 0.004) was more frequent in the bevacizumab group, and thromboembolic events occurred more often with bevacizumab (12.0% vs. 2.0%; *p* = 0.060), with grade 3/4 thrombosis significantly more frequent (10.0% vs. 0%; *p* = 0.027). Haematological toxicity, nausea and vomiting, fatigue and allergic or infusion reactions did not differ significantly between the groups (all *p* > 0.25); grade 3/4 neutropenia occurred in three patients (5.9%) in the anti-EGFR group and one patient (2.0%) in the bevacizumab group (*p* = 0.617).

## 4. Discussion

In this prospective single-centre cohort of 101 patients with RAS-wild-type colorectal cancer and initially unresectable liver metastases, first-line anti-EGFR therapy combined with a chemotherapy doublet was associated with a higher overall response rate (70.6% vs. 50.0%; *p* = 0.034), a deeper response (47.2% vs. 33.7% tumour shrinkage; *p* = 0.004) and a higher resection rate (66.7% vs. 46.0%; *p* = 0.036) than bevacizumab. Median PFS was significantly longer with anti-EGFR therapy (12.1 vs. 8.5 months; log-rank *p* = 0.016), and the association persisted after anadjustment for nine baseline covariates (HR 0.48, 95% CI 0.29–0.78; *p* = 0.003). Median OS was numerically longer in the anti-EGFR group (30.4 vs. 25.5 months), but the difference was not statistically significant (log-rank *p* = 0.382; adjusted HR 0.64, 95% CI 0.36–1.16; *p* = 0.141). Grade 3/4 adverse events did not differ significantly (35.3% vs. 22.0%; *p* = 0.187), although any-grade events were more frequent with anti-EGFR therapy (92.2% vs. 70.0%; *p* = 0.005), mainly because of skin toxicity.

The pattern of results is biologically coherent. When the aim of treatment is conversion, the speed and depth of tumour shrinkage are the main determinants of whether surgery becomes feasible, and anti-EGFR antibodies are known to produce early and deep responses in RAS-wild-type disease. Our findings are in line with pooled data showing higher response and resection rates, including R0 resection, with anti-EGFR therapy in RAS-wild-type conversion regimens [10,11]. The disease control rate did not differ (80.4% vs. 66.0%; *p* = 0.102), which suggests that the advantage of anti-EGFR therapy lies in the quality of response rather than in the proportion of patients whose disease is merely controlled.

The lack of a significant OS difference deserves careful interpretation. With 70 events, the study had limited power to detect an OS difference of the observed size, and the confidence interval of the adjusted HR (0.36–1.16) is compatible with both a substantial benefit and a small disadvantage. Furthermore, once metastases are resected, the long-term outcome probably depends more on the surgery itself than on the agent used to achieve it. Among the 57 operated patients, OS was similar between the groups (median 37.1 vs. 39.7 months; HR 1.06, 95% CI 0.56–2.03), whereas PFS showed a trend in favour of anti-EGFR therapy (14.4 vs. 11.0 months; HR 0.59, 95% CI 0.33–1.04; *p* = 0.065). This subgroup analysis is hypothesis-generating only, as it is restricted to a selected population of responders. The proportion of R1 resections was similar in both groups (20.6% vs. 21.7%), and R1 resection was not associated with worse PFS or OS (HR 0.66 and 0.91, respectively), although only 12 patients had R1 resection and the analysis was underpowered. Finally, the proportional hazards assumption was not fully satisfied for PFS (*p* = 0.026), and the absolute difference in progression-free proportions narrowed from 12 months (51.9% vs. 28.8%) to 24 months (10.0% vs. 4.1%); the reported HR should therefore be read as an average effect over the follow-up period.

Our results can be compared with the two randomised studies that addressed this question directly in unresectable colorectal liver metastases. In the ATOM trial [12], cetuximab was compared with bevacizumab, both combined with mFOLFOX6. The authors reported a significantly higher ORR with cetuximab (84.7% vs. 68.4%; *p* = 0.0483), but no difference in the resection rate (49.1% vs. 47.5%), PFS or OS. That trial also included patients with NRAS mutations, which confer resistance to anti-EGFR therapy and are associated with worse outcomes [13], and resectability was assessed after eight cycles, whereas in our study, surgery was considered from the fourth cycle onwards. Earlier re-evaluation probably favours the anti-EGFR group, which is characterised by early tumour shrinkage, and may partly explain the higher resection rate that we observed.

The phase 3 CAIRO5 trial [14] is the largest comparison to date. In patients with left-sided, RAS- and BRAF V600E-wild-type tumours, the addition of panitumumab to chemotherapy showed no advantage over bevacizumab in PFS, the local treatment rate or OS and was associated with more toxicity. This is an apparent contrast to our findings. CAIRO5 restricted this comparison to left-sided tumours, used a centralised expert-panel definition of unresectability that was re-evaluated throughout treatmentand allowed local ablative treatment in addition to surgery, all of which may have raised the threshold for demonstrating an anti-EGFR advantage in conversion. In addition, the BRAF and MSI status were not available in our cohort, whereas CAIRO5 included only BRAF V600E-wild-type tumours, so unrecognised differences in tumour biology between our groups may have influenced the comparison. In addition, in our study, the choice of the biological agent was not randomised, and the two groups differed in important prognostic characteristics, as discussed below. The anti-EGFR benefit seen in our cohort should therefore be interpreted with caution and confirmed in adequately powered studies. In contrast, the final analysis of the PARADIGM trial [15] forleft-sided RAS-wild-type mCRC showed significantly longer OS with panitumumab plus mFOLFOX6 than with bevacizumab (37.9 vs. 34.3 months; HR 0.82; *p* = 0.03), with the benefit emerging after approximately two years despite similar PFS and higher toxicity in the panitumumab arm. Together with FIRE-3 [16], CALGB/SWOG 80405 [17] and the pooled analysis by Arnold et al. [18], the available randomised evidence is therefore heterogeneous, and primary tumour sidedness is the most important modifier of the anti-EGFR benefit.

Tumour sidedness is the most obvious source of confounding in our cohort: 90.2% of patients in the anti-EGFR group had left-sided tumours, compared with 64.0% in the bevacizumab group (*p* = 0.002). Right-sided tumours have a worse prognosis and frequently harbour BRAF and other MAPK pathway alterations that confer intrinsic resistance to anti-EGFR therapy [19]. The imbalance may therefore have favoured the anti-EGFR group. In the multivariable model, however, sidedness was not significantly associated with PFS (HR 1.08, 95% CI 0.63–1.86) or OS (HR 1.76, 95% CI 0.87–3.54), and the effect of anti-EGFR therapy on PFS remained after an adjustment. Only five patients in the anti-EGFR group had right-sided tumours, so no conclusion about this subgroup is possible, and our results apply mainly to left-sided disease. In a sensitivity analysis restricted to left-sided tumours, the PFS advantage of anti-EGFR therapy persisted (adjusted HR 0.53, 95% CI 0.30–0.94), whereas OS did not differ significantly (adjusted HR 0.57, 95% CI 0.29–1.13). The groups also differed in the chemotherapy backbone, since FOLFIRI was used in 39.2% of anti-EGFR patients but in only 4.0% of bevacizumab patients. FOLFOX and FOLFIRI have shown similar efficacy in combination with biological agents [5,6,7,8,20,21], and the adjusted analysis with and without the backbone gave consistent results (PFS HR 0.48 and 0.53), but residual confounding cannot be excluded. Conversely, ECOG performance status 1 was more frequent in the anti-EGFR group (68.6% vs. 50.0%; *p* = 0.06), which would be expected to work against it.

The multivariable analysis also identified the tumour burden as the main prognostic factor: five or more liver metastases were associated with worse PFS (HR 1.72, 95% CI 1.03–2.88) and metastases larger than 5 cm with worse OS (HR 1.85, 95% CI 1.03–3.33), while extrahepatic disease showed a tendency towards shorter OS (HR 2.15, 95% CI 0.94–4.92). This supports the practice of selecting patients and the intensity of treatment according to the tumour burdenand underlines that a high-response regimen is most valuable forpatients with extensive disease, in whom resection depends on substantial shrinkage. The wide confidence intervals of several covariates reflect the limited number of events relative to the number of variables, and these estimates should be regarded as exploratory.

Within the anti-EGFR group, cetuximab and panitumumab produced similar responses (ORR 71.4% vs. 69.6%) and resection rates (60.7% vs. 73.9%; *p* = 0.381). Median PFS was numerically longer with panitumumab (12.9 vs. 12.1 months), and the unadjusted difference was statistically significant (HR 0.48, 95% CI 0.24–0.97; *p* = 0.037), but it disappeared after adjustment for the chemotherapy backbone (HR 0.56, 95% CI 0.21–1.53). Because all patients treated with panitumumab received FOLFOX, while 71.4% of those treated with cetuximab received FOLFIRI, the effect of the antibody cannot be separated from the effect of the backbone in this dataset. OS did not differ between the antibodies. These observations are consistent with a class effect of anti-EGFR antibodies, in line with the non-inferior OS of panitumumab versus cetuximab in the ASPECCT trial [22] and with real-world data in left-sided RAS wild-type mCRC [23], but our sample is too small for a formal comparison.

The safety profile reflected the mechanisms of action of the two drug classes. Skin rashes occurred in 86.3% of patients treated with anti-EGFR therapy and was grade 3/4 in 19.6%, and diarrhoea was more frequent (35.3% vs. 10.0%). Bevacizumab was associated with hypertension (38.0%), grade 3/4 thrombosis (10.0%) and polyneuropathy (20.0%). Part of these differences is probably explained by the chemotherapy backbone: oxaliplatin was used in 96.0% of bevacizumab-treated patients but in 60.8% of anti-EGFR patients, which may account for the higher frequency of polyneuropathy in the bevacizumab group, while the greater use of irinotecan in the anti-EGFR group may have contributed to diarrhoea. Since the rates of grade 3/4 events were not significantly different and skin toxicity is generally predictable and can be managed with pre-emptive measures, the toxicity of anti-EGFR therapy should not preclude its use when conversion is the goal. However, adverse events were based on patient self-reports, which may have led to the under-recording of asymptomatic laboratory abnormalities.

This study has several limitations. It was a single-centre study with a modest sample size, and patients were allocated to the treatment groups in an alternating manner rather than randomly, so selection bias and confounding by indication cannot be excluded. The groups were not balanced with respect to sidedness and the chemotherapy backbone, the choice of anti-EGFR agent depended on availability and the BRAF, MSI and HER2 status were not available. We did not collect data on subsequent lines of therapy, which may have affected OS. The number of events limited the number of covariates that could be included in the multivariable models, the proportional hazards assumption was not fully met for PFSand the analyses in operated patients and in the cetuximab and panitumumab subgroups were underpowered and exploratory. Finally, the toxicity data were based on self-reporting. The strengths of the study include its prospective design, central RAS testing, resectability assessment by two independent hepatobiliary surgeons repeated every four cycles and a long median follow-up of 59.4 months, which allowed a mature analysis of survival.

Triplet regimens such as FOLFOXIRI combined with bevacizumab [24] or panitumumab [25] are an alternative conversion strategy for patients with a high tumour burden, at the cost of greater toxicity; they were not used in our cohort. Looking forward, the consensus molecular subtypes of colorectal cancer have prognostic and predictive value: CMS4 tumours seem to benefit more from irinotecan-based regimens, CMS1 tumours from the addition of bevacizumab and RAS-wild-type CMS2 tumours from anti-EGFR agents [26]. Incorporating molecular classification, together with sidedness, and the BRAF, MSI and HER2 status into the selection of a conversion regimen could further improve outcomes. Beyond the choice of the biological agent, new delivery strategies, such as nanoparticle-based systems, are being investigated to improve the efficacy and tolerability of systemic treatment in colorectal cancer [27]. Frameworks that integrate efficacy and safety into a single measure, such as ESIRA proposed for the first-line treatment of hepatocellular carcinoma [28], may also help to compare regimens with different toxicity profiles, as in the present study. Non-invasive approaches, such as CT radiomics for predicting theKRAS, NRAS and BRAF mutation status [29], may, in the future, complement tissue-based molecular testing.

## 5. Conclusions

In patients with RAS-wild-type colorectal cancer and initially unresectable liver metastases, first-line anti-EGFR therapy combined with chemotherapy was associated with a higher response rate, a higher resection rate and longer progression-free survival than bevacizumab, with no significant difference in overall survival and in the rate of grade 3/4 adverse events. These findings suggest that anti-EGFR therapy may be considered when conversion to resection is the goal, particularly in patients with left-sided primary tumours. As real-world evidence from a single centre, with a non-randomised design and an imbalance in prognostic factors, these findings are hypothesis-generating and do not replace randomised trials; they should be confirmed in adequately powered prospective studies.

## Figures and Tables

**Table 1:** Baseline demographic, clinical and treatment characteristics of patients treated with anti-EGFR agents versus bevacizumab.

*p*	Bevacizumab Group	Anti-EGFR Group			
0.94	33 (66.0)	34 (66.7)	Men	Sex, *n* (%)	
	17 (34.0)	17 (33.3)	Women		
0.97	60.56 ± 9.88	60.65 ± 10.75	Age, mean ± SD		
0.06	25 (50.0)	16 (31.4)	ECOG 0	Performance status, *n* (%)	
	25 (50.0)	35 (68.6)	ECOG 1		
0.77	29 (58.0)	31 (60.8)	Comorbidity, *n* (%)		
0.002	32 (64.0)	46 (90.2)	Left-sided		Sidedness, *n* (%)
	18 (36.0)	5 (9.8)	Right-sided		
0.36	32 (64.0)	37 (72.5)	1–4		Number of hepatic metastases, *n* (%)
	18 (36.0)	14 (27.5)	≥5		
0.13	63 ± 32.77	73.51 ± 37.17	Sum of diameters of target lesions, mean ± SD		
0.49	19 (38.0)	16 (31.4)	≤5 cm	Diameter of metastases, *n* (%)	
	31 (62.0)	35 (68.6)	>5 cm		
0.85	39 (78.0)	39 (76.5)	Synchronous	Appearance of metastases, *n* (%)	
	11 (22.0)	12 (23.5)	Metachronous		
0.56	5 (10.0)	7 (13.7)	Other organs involved, *n* (%)		
<0.001	2 (4.0)	20 (39.2)	FOLFIRI	Chemotherapy, *n* (%)	
	48 (96.0)	31 (60.8)	FOLFOX		
0.10	6.74 ± 3.01	5.88 ± 2.11	Number of preoperative therapy cycles, mean ± SD		

ECOG, Eastern Cooperative Oncology Group; SD, standard deviation; FOLFOX, folinic acid, fluorouracil and oxaliplatin; FOLFIRI, folinic acid, fluorouracil and irinotecan. Values are *n* (%) or the mean ± SD. *p*-values were calculated using the chi-squared or Fisher’s exact test for categorical variables and Student’s *t*-test or the Mann–Whitney U test for continuous variables.

**Table 2:** Treatment response and liver resection rates in patients treated with anti-EGFR agents versus bevacizumab.

*p*	Bevacizumab	Anti-EGFR Group	
0.034	25 (50.0)	36 (70.6)	Overall response rate, *n* (%)
0.102	33 (66.0)	41 (80.4)	Disease control rate, *n* (%)
0.004	33.67 ± 25.27	47.20 ± 22.79	Depth of response, %, mean ± SD
0.036	23 (46.0)	34 (66.7)	Resection rate, *n* (%)
1.000	18 (78.3)	27 (79.4)	R0 resection
1.000	5 (21.7)	7 (20.6)	R1 resection

SD, standard deviation. Depth of response is the maximal percentage decrease in the sum of diameters of target lesions (RECIST 1.1). R0 and R1, resection with microscopically negative and positive margins, respectively; percentages for R0 and R1 resection are calculated among operated patients (*n* = 34 and *n* = 23). *p*-values were calculated using the chi-squared or Fisher’s exact test for categorical variables and Student’s *t*-test for depth of response.

**Table 4:** Cetuximab versus panitumumab: patient characteristics, response and survival.

*p*	Panitumumab (*n* = 23)	Cetuximab (*n* = 28)	
Patient and tumour characteristics			
0.039	19 (82.6)	15 (53.6)	Male sex, *n* (%)
0.156	62.9 ± 7.7	58.8 ± 12.6	Age, years, mean ± SD
0.552	17 (73.9)	18 (64.3)	ECOG 1, *n* (%)
1.000	14 (60.9)	17 (60.7)	Comorbidity, *n* (%)
1.000	2 (8.7)	3 (10.7)	Right-sided tumour, *n* (%)
1.000	6 (26.1)	8 (28.6)	≥5 liver metastases, *n* (%)
0.659	71.0 ± 32.2	75.6 ± 41.3	Sum of target lesion diameters, mm, mean ± SD
1.000	16 (69.6)	19 (67.9)	Metastases > 5 cm, *n* (%)
0.107	8 (34.8)	4 (14.3)	Metachronous metastases, *n* (%)
1.000	4 (17.4)	5 (17.9)	Other organs involved, *n* (%)
<0.001	0 (0.0)	20 (71.4)	FOLFIRI backbone, *n* (%)
Treatment			
0.292	6.9 ± 2.8	6.1 ± 2.5	Therapy cycles, mean ± SD
Response and resection			
1.000	16 (69.6)	20 (71.4)	Overall response rate, *n* (%)
1.000	19 (82.6)	22 (78.6)	Disease control rate, *n* (%)
0.475	50.0 ± 24.1	44.8 ± 21.8	Depth of response, %, mean ± SD †
0.381	17 (73.9)	17 (60.7)	Resection rate, *n* (%)
Survival			
0.037 ‡	12.9 (9.6–20.3)	12.1 (8.1–14.4)	Median PFS, months (95% CI)
0.141 ‡	32.0 (20.4–48.2)	27.5 (20.5–37.1)	Median OS, months (95% CI)

ECOG, Eastern Cooperative Oncology Group; SD, standard deviation; PFS, progression-free survival; OS, overall survival; CI, confidence interval; FOLFIRI, folinic acid, fluorouracil and irinotecan. Values are *n* (%) or mean ± SD unless stated otherwise. The patient who received cetuximab followed by panitumumab is included in the panitumumab group. Categorical variables were compared with Fisher’s exact test and means with Welch’s *t*-test. † Calculated in patients with disease control (*n* = 22 and *n* = 19). ‡ Log-rank test. Hazard ratio (panitumumab vs. cetuximab) for PFS: 0.48 (95% CI 0.24–0.97) unadjusted and 0.56 (95% CI 0.21–1.53) adjusted for chemotherapy backbone; for OS: 0.57 (95% CI 0.27–1.21) unadjusted and 0.57 (95% CI 0.19–1.66) adjusted.

**Table 5:** Patients with any and grade 3/4 adverse events.

*p*	Bevacizumab (*n* = 50)	Anti-EGFR (*n* = 51)	
0.005	35 (70.0)	47 (92.2)	Any adverse event, *n* (%)
0.187	11 (22.0)	18 (35.3)	Grade 3/4 adverse event, *n* (%)

EGFR, epidermal growth factor receptor. Adverse events were graded according to CTCAE version 5.0; grade 3/4 indicates at least one grade 3 or 4 adverse event. Each patient is counted once, irrespective of the number of adverse events. *p*-values were calculated using Fisher’s exact test.

**Table 6:** Adverse events in patients treated with anti-EGFR therapy and bevacizumab, *n* (%).

Grade 3/4			All Grades			
*p*	Bevacizumab (*n* = 50)	Anti-EGFR (*n* = 51)	*p*	Bevacizumab (*n* = 50)	Anti-EGFR (*n* = 51)	Adverse Event
0.001	0 (0.0)	10 (19.6)	<0.001	1 (2.0)	44 (86.3)	Rash
0.495	0 (0.0)	2 (3.9)	0.004	5 (10.0)	18 (35.3)	Diarrhoea
1.000	0 (0.0)	1 (2.0)	0.056	0 (0.0)	5 (9.8)	Stomatitis
1.000	0 (0.0)	0 (0.0)	0.243	0 (0.0)	3 (5.9)	Hand-foot syndrome
1.000	0 (0.0)	0 (0.0)	0.495	0 (0.0)	2 (3.9)	Paronychia
0.056	4 (8.0)	0 (0.0)	<0.001	19 (38.0)	0 (0.0)	Hypertension
0.027	5 (10.0)	0 (0.0)	0.060	6 (12.0)	1 (2.0)	Thrombosis
0.118	3 (6.0)	0 (0.0)	0.004	10 (20.0)	1 (2.0)	Polyneuropathy
0.617	1 (2.0)	3 (5.9)	1.000	4 (8.0)	4 (7.8)	Neutropenia
0.495	1 (2.0)	0 (0.0)	0.118	3 (6.0)	0 (0.0)	Thrombocytopenia
1.000	0 (0.0)	0 (0.0)	0.625	11 (22.0)	9 (17.6)	Nausea/vomiting
1.000	0 (0.0)	0 (0.0)	0.269	2 (4.0)	6 (11.8)	Fatigue
0.617	1 (2.0)	3 (5.9)	0.362	1 (2.0)	4 (7.8)	Allergy/infusion reaction

EGFR, epidermal growth factor receptor. Adverse events were graded according to CTCAE version 5.0. Percentages are calculated within each treatment group, and each patient is counted once per adverse event. *p*-values were calculated using Fisher’s exact test.

## Data Availability

The data presented in this study are available on request from the corresponding author due to privacy and ethical restrictions.

## Supplementary Materials

The following supporting information can be downloaded at: https://www.mdpi.com/article/10.3390/cancers18213489/s1. Table S1: Exploratory subgroup analyses of response, resection and survival: anti-EGFR therapy vs. bevacizumab; Figure S1: Forest plot of progression-free survival by subgroup (anti-EGFR therapy vs. bevacizumab).; Figure S2: Forest plot of overall survival by subgroup (anti-EGFR therapy vs. bevacizumab); Table S2: Progression after liver resection and site of first progression in operated patients. Table S3: Sensitivity analysis restricted to patients with left-sided primary tumours (*n* = 78) compared with the main analysis; Table S4: Efficacy by chemotherapy backbone in patients treated with anti-EGFR therapy (*n* = 51).

## Abbreviations

The following abbreviations are used in this manuscript:

mCRC	metastatic colorectal cancer
CRLM	colorectal liver metastases
VEGF	vascular endothelial growth factor
EGFR	epidermal growth factor receptor
ORR	overall response rate
DCR	disease control rate
DoR	depth of response
RR	resection rate
R0	resection without microscopically positive margins
R1	resection with microscopically positive margins
PFS	progression-free survival
mPFS	median progression-free survival
OS	overall survival
mOS	median overall survival
ECOG	Eastern Cooperative Oncology Group
MAPK	mitogen-activated protein kinase
MSCT	computed tomography
RECIST	Response Evaluation Criteria in Solid Tumours
CR	complete response
PR	partial response
SD	stable disease; standard deviation (in tables)
ULN	upper limit of normal
AST	aspartate aminotransferase
ALT	alanine aminotransferase
CTCAE	Common Terminology Criteria for Adverse Events
FOLFOX	folinic acid, fluorouracil and oxaliplatin
FOLFOXIRI	folinic acid, fluorouracil, oxaliplatin and irinotecan
FOLFIRI	folinic acid, fluorouracil and irinotecan
CI	confidence interval
HR	hazard ratio
CMS	consensus molecular subtype
MSI	microsatellite instability
