# Supplementary material for: The Optimal Biological Therapy for Conversion in Patients with RAS-Wild-Type Colorectal Cancer and Unresectable Liver Metastases
Source: Cancers (Basel). 2026 Oct 29;18(21):3489. doi: 10.3390/cancers18213489 (PMC13762230; doi:10.3390/cancers18213489)
Supplement: Supplementary file 1 [file cancers-18-03489-s001.zip › cancers-4655763-supplementary.pdf]

Supplementary Materials

The optimal biological therapy for conversion in patients with RAS wild-type colorectal cancer and unresectable liver metastases

Contents: Table S1, exploratory subgroup analyses; Figure S1 and Figure S2, forest plots of progression-free and overall survival by subgroup; Table S2, sensitivity analysis in left-sided tumours; Table S3, efficacy by chemotherapy backbone in the anti-EGFR group; Table S4, progression after liver resection.

Table S1. Exploratory subgroup analyses of response, resection and survival: anti-EGFR therapy vs. bevacizumab.

Subgroup	n (anti-EGFR / bevacizumab)	ORR, % (p)	Resection rate, % (p)	PFS HR (95% CI)	p interaction, PFS	OS HR (95% CI)	p interaction, OS
**Sex**							
**Female**	17 / 17	58.8 vs. 29.4 (p=0.166)	58.8 vs. 29.4 (p=0.166)	0.41 (0.19–0.88)	0.196	0.75 (0.33–1.71)	0.957
**Male**	34 / 33	76.5 vs. 60.6 (p=0.194)	70.6 vs. 54.5 (p=0.212)	0.66 (0.39–1.12)		0.77 (0.42–1.41)	
**Age**							
**≤50 years**	9 / 5	55.6 vs. 60.0 (p=1.000)	55.6 vs. 40.0 (p=1.000)	1.17 (0.36–3.84)	0.224	2.04 (0.42–9.99)	0.198
**>50 years**	42 / 45	73.8 vs. 48.9 (p=0.027)	69.0 vs. 46.7 (p=0.051)	0.53 (0.33–0.85)		0.71 (0.42–1.20)	
**ECOG PS**							
**ECOG 0**	16 / 25	87.5 vs. 44.0 (p=0.008)	87.5 vs. 28.0 (p<0.001)	0.49 (0.24–1.00)	0.372	0.34 (0.15–0.81)	0.011
**ECOG 1**	35 / 25	62.9 vs. 56.0 (p=0.606)	57.1 vs. 64.0 (p=0.790)	0.71 (0.41–1.23)		1.33 (0.70–2.51)	
**Sidedness**							
**Left-sided**	46 / 32	69.6 vs. 53.1 (p=0.159)	67.4 vs. 53.1 (p=0.241)	0.61 (0.38–0.99)	0.931	0.85 (0.50–1.45)	0.650
**Right-sided**	5 / 18	80.0 vs. 44.4 (p=0.317)	60.0 vs. 33.3 (p=0.343)	0.61 (0.20–1.88)		1.22 (0.32–4.70)	
**Liver metastases**							
**1–4 metastases**	37 / 32	70.3 vs. 53.1 (p=0.213)	67.6 vs. 46.9 (p=0.094)	0.70 (0.42–1.17)	0.196	0.86 (0.49–1.51)	0.601
**≥5 metastases**	14 / 18	71.4 vs. 44.4 (p=0.165)	64.3 vs. 44.4 (p=0.308)	0.35 (0.13–0.91)		0.61 (0.23–1.63)	
**Metastasis size**							
**≤5 cm**	16 / 19	62.5 vs. 31.6 (p=0.095)	62.5 vs. 36.8 (p=0.181)	0.79 (0.38–1.63)	0.389	0.87 (0.37–2.03)	0.700
**>5 cm**	35 / 31	74.3 vs. 61.3 (p=0.298)	68.6 vs. 51.6 (p=0.209)	0.51 (0.30–0.87)		0.67 (0.37–1.22)	
**Extrahepatic disease**							
**No extrahepatic disease**	42 / 45	76.2 vs. 51.1 (p=0.025)	69.0 vs. 48.9 (p=0.081)	0.61 (0.39–0.96)	0.630	0.81 (0.49–1.35)	0.200
**Extrahepatic disease**	9 / 5	44.4 vs. 40.0 (p=1.000)	55.6 vs. 20.0 (p=0.301)	0.47 (0.12–1.76)		0.61 (0.12–3.08)	
**Timing of metastases**							
**Synchronous**	39 / 39	71.8 vs. 48.7 (p=0.063)	66.7 vs. 41.0 (p=0.040)	0.57 (0.35–0.92)	0.828	0.76 (0.43–1.34)	0.613
**Metachronous**	12 / 11	66.7 vs. 54.5 (p=0.680)	66.7 vs. 63.6 (p=1.000)	0.64 (0.26–1.61)		0.98 (0.39–2.43)	
**Chemotherapy**							
**FOLFOX**	31 / 48	67.7 vs. 50.0 (p=0.163)	71.0 vs. 45.8 (p=0.037)	0.47 (0.28–0.78)	0.137	0.69 (0.39–1.23)	0.385
**FOLFIRI**	20 / 2	75.0 vs. 50.0 (p=0.481)	60.0 vs. 50.0 (p=1.000)	1.67 (0.37–7.62)		1.55 (0.33–7.20)	

Hazard ratios (HR) for anti-EGFR therapy vs. bevacizumab are unadjusted (Cox proportional hazards model, Efron method for ties). p interaction was obtained from a Cox model containing treatment, the subgroup variable and their product. ORR, overall response rate; PFS, progression-free survival; OS, overall survival; CI, confidence interval; n.e., not estimable. Response and resection rates were compared with Fisher's exact test within each subgroup. No correction for multiple comparisons was made; all analyses are exploratory and hypothesis-generating. Age was dichotomised at 50 years.

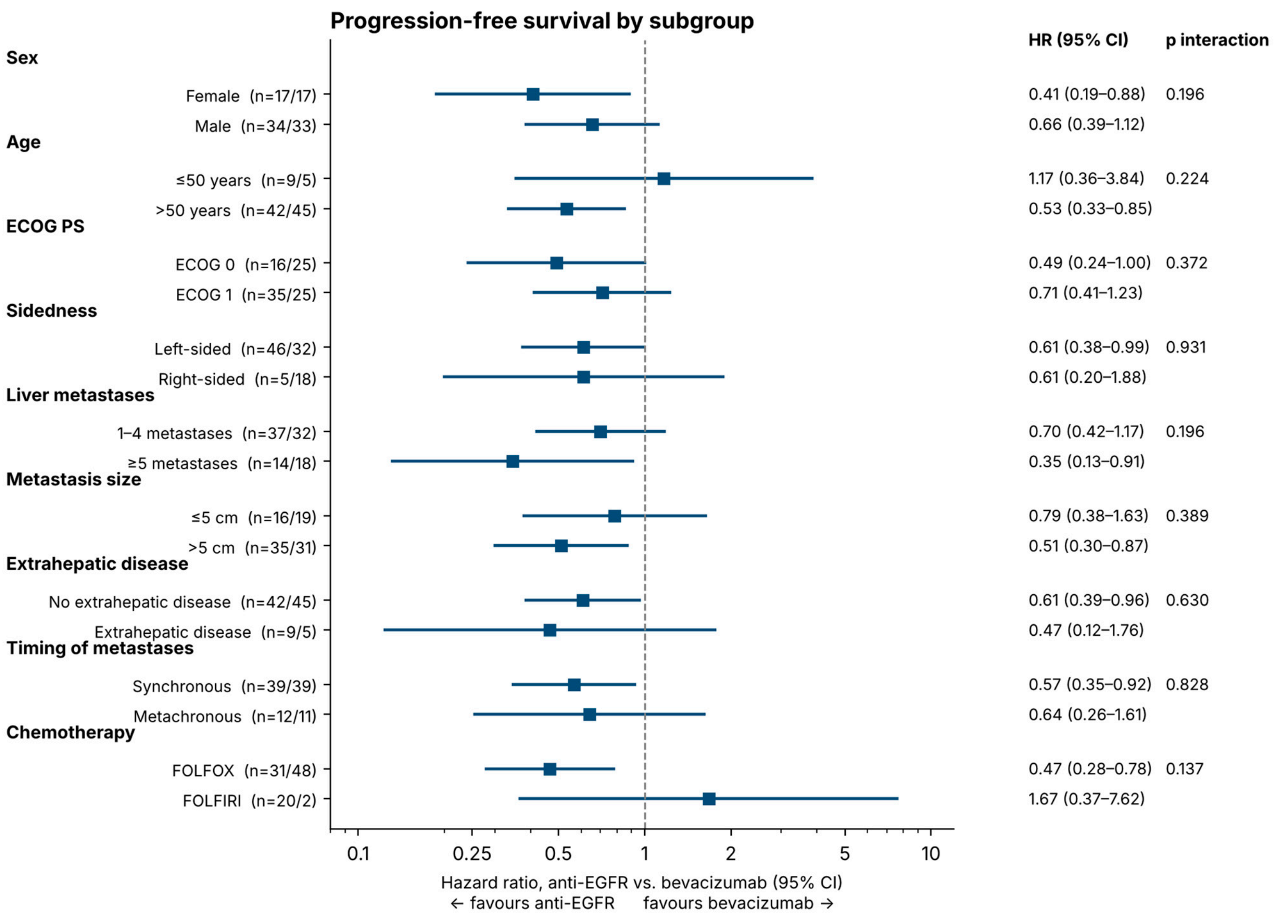

Figure S1. Forest plot of progression-free survival by subgroup (anti-EGFR therapy vs. bevacizumab). Squares are unadjusted hazard ratios and horizontal lines 95% confidence intervals; the dashed line indicates HR = 1 and values below 1 favour anti-EGFR therapy. Numbers in brackets are patients in the anti-EGFR and bevacizumab groups. p interaction is from a treatment-by-subgroup Cox model. The analyses are exploratory and not corrected for multiple comparisons; estimates for small subgroups are imprecise.

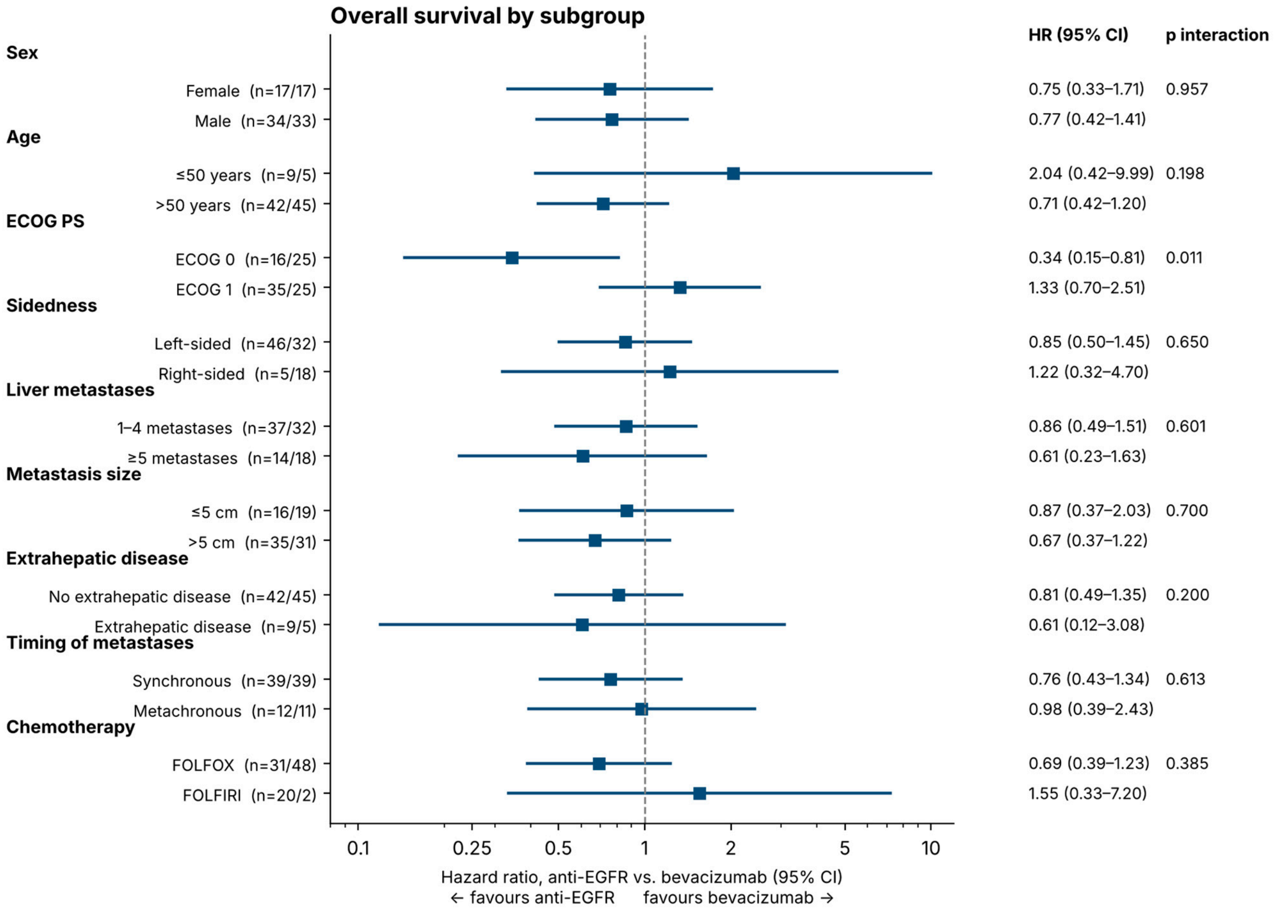

Figure S2. Forest plot of overall survival by subgroup (anti-EGFR therapy vs. bevacizumab). Squares are unadjusted hazard ratios and horizontal lines 95% confidence intervals; the dashed line indicates HR = 1 and values below 1 favour anti-EGFR therapy. Numbers in brackets are patients in the anti-EGFR and bevacizumab groups. p interaction is from a treatment-by-subgroup Cox model. The analyses are exploratory and not corrected for multiple comparisons; estimates for small subgroups are imprecise.

Table S2. Progression after liver resection and site of first progression in operated patients.

	Anti-EGFR	Bevacizumab	p
**Operated patients, n**	34	23	
**Progression or recurrence, n (%)**	27 (79.4)	23 (100)	0.034
**No progression at last follow-up, n (%)**	7 (20.6)	0 (0)	
**Site of first progression among patients with a recorded site (n = 22 and n = 20)**			
**Liver only, n (%)**	16 (72.7)	13 (65.0)	
**Extrahepatic only, n (%)**	1 (4.5)	3 (15.0)	
**Liver and extrahepatic, n (%)**	5 (22.7)	4 (20.0)	
**Site not recorded, n**	5	3	

p-value: Fisher's exact test. Extrahepatic sites included lung, bone, pleura, lymph nodes, skin, peritoneum, mesentery and retroperitoneum. Progression refers to radiological progression or recurrence at any time after the start of treatment. The date of surgery and any treatment of recurrence (repeat resection, ablation) were not recorded, so recurrence-free survival from the date of surgery could not be calculated.

Table S3. Sensitivity analysis restricted to patients with left-sided primary tumours (n = 78) compared with the main analysis.

Endpoint	All patients (anti-EGFR vs. bevacizumab)	Left-sided tumours only (anti-EGFR vs. bevacizumab)
**ORR, n (%)**	36 (70.6) vs. 25 (50.0); p=0.034	32 (69.6) vs. 17 (53.1); p=0.159
**DCR, n (%)**	41 (80.4) vs. 33 (66.0); p=0.102	37 (80.4) vs. 22 (68.8); p=0.288
**Depth of response, % (mean ± SD)**	47.2 ± 22.8 vs. 33.7 ± 25.3; p=0.004	47.4 ± 23.4 vs. 32.5 ± 19.6; p=0.011
**Resection rate, n (%)**	34 (66.7) vs. 23 (46.0); p=0.036	31 (67.4) vs. 17 (53.1); p=0.241
**Median PFS, months**	12.1 vs. 8.5; log-rank p=0.016	12.2 vs. 9.1; log-rank p=0.044
**PFS HR, unadjusted**	0.60 (0.39–0.91)	0.61 (0.38–0.99)
**PFS HR, adjusted***	0.48 (0.29–0.78); p=0.003	0.53 (0.30–0.94); p=0.031
**PFS HR, adjusted without chemotherapy backbone**	0.53 (0.34–0.84); p=0.006	0.57 (0.35–0.95); p=0.030
**Median OS, months**	30.4 vs. 25.5; log-rank p=0.382	32.0 vs. 26.0; log-rank p=0.566
**OS HR, unadjusted**	0.81 (0.50–1.30)	0.85 (0.50–1.45)
**OS HR, adjusted***	0.64 (0.36–1.16); p=0.141	0.57 (0.29–1.13); p=0.107
**OS HR, adjusted without chemotherapy backbone**	0.71 (0.42–1.21); p=0.206	0.70 (0.40–1.23); p=0.213
**Patients, anti-EGFR / bevacizumab**	51 / 50	46 / 32
**Events, PFS / OS**	89 / 70	69 / 56

HR, hazard ratio (95% CI) for anti-EGFR therapy vs. bevacizumab. * Adjusted for age, sex, ECOG performance status, number and size of liver metastases, extrahepatic disease, timing of metastases and chemotherapy backbone (and tumour sidedness in the analysis of all patients). Sidedness was not included in the left-sided analysis because it was constant. ORR, overall response rate; DCR, disease control rate; PFS, progression-free survival; OS, overall survival; SD, standard deviation.

Table S4. Efficacy by chemotherapy backbone in patients treated with anti-EGFR therapy (n = 51).

**Measure**	**FOLFOX (n = 31)**	**FOLFIRI (n = 20)**	**p**
**ORR, n (%)**	21 (67.7)	15 (75.0)	0.755
**DCR, n (%)**	25 (80.6)	16 (80.0)	1.000
**Depth of response, % (mean ± SD)†**	49.1 ± 24.9	44.2 ± 19.4	0.482
**Resection rate, n (%)**	22 (71.0)	12 (60.0)	0.545
**Median PFS, months (events)**	12.6 (24)	11.7 (17)	0.057
**Median OS, months (events)**	32.0 (18)	27.5 (14)	0.268

**Hazard ratio, FOLFIRI vs. FOLFOX**	PFS HR (95% CI); p	OS HR (95% CI); p
**Unadjusted**	1.88 (0.97–3.65); p=0.060	1.51 (0.73–3.13); p=0.268
**Adjusted‡**	1.57 (0.57–4.31); p=0.383	1.61 (0.40–6.52); p=0.501

p-values: Fisher's exact test (proportions), Welch's t-test (depth of response), log-rank test (median PFS and OS), Wald test (hazard ratios). † Patients with an evaluable response (n = 25 and n = 16). ‡ Adjusted for ECOG performance status, tumour sidedness, number of liver metastases and anti-EGFR agent. All panitumumab-treated patients received FOLFOX, so the backbone and the antibody are partly confounded. ORR, overall response rate; DCR, disease control rate; PFS, progression-free survival; OS, overall survival.
